# Comparative analysis prediction of prostate and testicular cancer mortality using machine learning: accuracy study

**DOI:** 10.1590/1516-3180.2024.0080.03072024

**Published:** 2025-02-24

**Authors:** Aurélio Gomes de Albuquerque, David Medeiros Nery, João Paulo Araújo Braz, Carla Ferreira do Nascimento, Tiago Almeida de Oliveira, Brígida Gabriele Albuquerque Barra, Leonardo Thiago Duarte Barreto Nobre, Diego Bonfada, Janine Karla França da Silva Braz

**Affiliations:** I Escola Multicampi de Ciências Médicas do Rio Grande do Norte, Universidade Federal do Rio Grande do Norte (UFRN), Caicó (RN), Brazil.; II Escola Multicampi de Ciências Médicas do Rio Grande do Norte, Universidade Federal do Rio Grande do Norte (UFRN), Caicó (RN), Brazil.; III Pharmaceutical, Information analyst, Department of Central Pharmacy, Hospital Giselda Trigueiro, Natal (RN), Brazil.; IV Universidade do Estado da Bahia (UNEB), Salvador (BA), Brazil.; V Statistical and Associate Professor, Departament of Statistic, Universidade Estadual da Paraíba (UEPB), Campina Grande (PB), Brazil.; VI Postgraduate Program in Collective Health, Universidade Federal do Rio Grande do Norte (UFRN), Natal (RN), Brazil.; VII Adjunct Professor, Escola Multicampi de Ciências Médicas do Rio Grande do Norte, Universidade Federal do Rio Grande do Norte (UFRN), Caicó (RN), Brazil.; VIII Adjunct Professor, Escola Multicampi de Ciências Médicas do Rio Grande do Norte, Universidade Federal do Rio Grande do Norte (UFRN), Caicó (RN), Brazil.; IX Adjunct Professor, Escola Multicampi de Ciências Médicas do Rio Grande do Norte, Universidade Federal do Rio Grande do Norte (UFRN), Caicó (RN), Brazil.

**Keywords:** Prostatic neoplasm, Testicular neoplasm, Artificial intelligence, Protastic cancer, Testicular cancer, Python library

## Abstract

**BACKGROUND:**

The mortality rates of prostate and testicular cancer are higher mortality in the northeast region.

**OBJECTIVE:**

We aimed to compare the efficacy of machine learning libraries in predicting testicular and prostate cancer mortality.

**DESIGN AND SETTING:**

A comparative analysis of the pyMannKendall and Prophet machine-learning algorithms was conducted to develop predictive models using data from DATASUS (TabNet) to Caicó (Brazil) and Rio Grande do Norte (Brazil).

**METHODS:**

Data on prostate and testicular cancer mortality in men from 2000 to 2019 were collected. The prediction accuracy of the Prophet algorithm was evaluated using the mean squared error (MSE), the root mean squared error and analyzed using the pyMannKendall, and Prophet libraries.

**RESULTS:**

The research data were made publicly available on GitHub. The machine test confirmed the accuracy of the predictions, with the root MSE (RMSE) values closely matching the observed data for Caicó (RMSE = 2.46) and Rio Grande do Norte (RMSE = 22.85). The Prophet algorithm predicted an increase in prostate cancer mortality by 2030 in Caicó and Rio Grande do Norte. This prediction was corroborated by the pyMannKendall analysis, which indicated a 99% probability of a rising mortality trend in Caicó (P < 0.01; tau = 0.586; intercept = 2.59) and Rio Grande do Norte (P = 2.06; tau = 0.84, and intercept = 119.63). For testicular cancer, no significant mortality trend was identified by Prophet or pyMann-Kendall.

**CONCLUSIONS:**

Libraries are reliable tools for predicting mortality, providing support for strategic health planning, and implementing preventive measures to ensure men’s health. Addressing the gender gap in DATASUS is essential.

## INTRODUCTION

Cancer mortality is influenced by socioeconomic factors and exposure to risk factors, including lifestyle and social conditions, all of which are determinants of disease probability.^1^ The National Cancer Institute (INCA) projected 625,000 patients with newly diagnosed cancer in Brazil for the period 2020–2023, with prostate cancer being the second most prevalent, affecting 66,000 individuals.^2^

Prostate cancer is one of the leading causes of cancer-related mortality in Brazil, primarily affecting cisgender men aged 50 years and older.^3^ By contrast, testicular cancer, though considered rare, affects cisgender men aged 15–39 years, leading to significant social and reproductive consequences particularly within the economically active population.^3^ These cancer types are not exclusive to cisgender patients; transgender and transvestite women are also at risk.^4^ The use of hormones by these women may increase their susceptibility to developing reproductive cancers, including prostate and testicular cancers.^4^ However, due to the barriers to accessing healthcare, social discrimination, and the lack of appropriate guidance, reports of such cases remain scarce.^5^

In Brazil, the National Policy for Integral Attention to Men’s Health has been implemented to promote health education among men, addressing social, cultural, political, and economic aspects while respecting regional differences.^6^ However, the trends in cancer incidence and mortality, such as testicular and prostate cancer, exhibit regional disparities, with notably higher mortality rates in the northeast region.^7^ These disparities are influenced by factors beyond geographical location. Age, ethnicity, diet, family history, obesity, cigarette smoking, genetic factors, gender identity, lifestyle, and the availability of screening tests are all risk factors that increase the likelihood of developing a disease or health problem and affect the understanding of prostate cancer mortality.^8^ In addition, testicular cancer screening is less developed compared with prostate cancer screening.

Machine learning (ML) algorithms, a prominent subfield of artificial intelligence (AI), have been developed and utilized to analyze medical datasets since their initial development.^9^ This innovation enhances epidemiology as it optimizes the analysis of large data from multicenter databases, such as the Mortality Information System (SIM), thereby improving the quality and transparency of information.^10^ However, no study has employed these tools in the R and Python languages. ML is widely used for prediction in various fields, including primary healthcare, where it can forecast testosterone deficiency without requiring expensive medical tests.^11^ Additionally, ML has demonstrated its value in clinical prediction, facilitating diagnostic decision-making for conditions like aggressive breast cancer^12^ and predicting cancer risk, susceptibility, and recurrence across multiple types, such as lung, colorectal, esophageal, prostate, stomach, and thyroid.^13^

Splitting data into training and test sets is a standard practice in predictive analytics.^14^ Configuration selection is a critical aspect in tuning machine learning models, as overfitting can significantly impact the performance of different learning algorithms and must be carefully addressed in empirical evaluations.^15^ Various machine learning techniques, such as Prophet Library and pyMannKendall, are already being used for predictive analysis across different pathologies. In the clinical field, machine learning has been applied to address orthopedic issues, such as predicting the advancement of articular cartilage degeneration in chronic osteoarthrosis^16^ and forecasting the relative risk of dengue transmission in different locations.^17^

The Prophet library, an open-source forecasting model developed by Facebook based on ML techniques, automates parameter selection. This feature allows users to easily adjust model parameters to best fit the input data.^15^ It was designed to be a practical and accessible tool for time series forecasting.^18^ This enables Prophet’s forecasts customizable for non-experts and adaptable to the specific needs of health analysis, such as predicting the progression of coronavirus disease 2019 (COVID-19) cases in hospital intensive care units (ICUs).^19^ Intuitive parameter customization, such as smoothing for trend and seasonality, and the integration of prior information for growth curve boundaries, makes it possible to tailor the model to the specific needs of each analysis. This enhances the completeness and accuracy of health data analysis.

Despite its common use in environmental studies for analyzing the temporal and seasonal trends, pyMannKendall has not been widely applied in the health sector, particularly in the analysis of prostate and testicular cancer mortality.^20^ Google Colab, a platform that utilizes Python language for code execution and analysis, was effectively employed for breast cancer mortality analysis owing to its ease of use and the ability to rapidly integrate various Python libraries.^21^

## OBJECTIVES

We aimed to assess the accuracy of machine learning in predicting prostate and testicular cancer mortality in Rio Grande do Norte (RN) and Caicó, Brazil. The data and findings were made publicly available on GitHub (https://github.com/jpbraz/nanomed-colab-prophet-googlesheets). We also aimed to compare Prophet and pyManKendall tools to optimize mortality predictions in both regions, refining the quality of the results.

## METHODS

### Data

Data were collected from the TabNet platform, integrated into DATASUS, and publicly accessible (https://datasus.saude.gov.br/informacoes-de-saude-tabnet). Thus, this study uses public data and therefore does not require approval by an Ethics Committee. The “1996 International Classification of Diseases, Tenth Revision (ICD-10) mortality data” were used, selecting “general mortality” for RN and Caicó. We focused on the ICD-10 categories C61 (malignant prostate neoplasm) and C62 (malignant neoplasm of the testes) for the period 2000 and 2019, specifically for the male population. The tables were exported to CSV format and made available on Google Spreadsheets (https://docs.google.com/spreadsheets). Additionally, we collected the population data by age group for Caicó, RN, and Brazil to calculate the crude and age-adjusted prostate and testicular cancer-specific mortality rates. We used a direct method with the Brazilian population serving as the reference(**Table 1**).

**Table 1:** Prostate and testicular cancer mortality rates in the municipality of Caicó and in the state of Rio Grande do Norte from 2000 to 2019

Year	Caicó				Rio Grande do Norte			
	Population	Number of deaths	Crude mortality rate/100,000 pop.	Age adjusted mortality rate/100,000 pop.	Population	Number of deaths	Crude mortality rate/100,000 pop.	Age adjusted mortality rate/100,000 pop.
2000	58,594	3	5.1	4.1	2.853,035	74	2,6	2.3
2001	59,217	3	5.1	2.3	2,896,569	90	3.1	2.8
2002	59,808	5	8.4	6.3	2,937,858	97	3.3	2.8
2003	60,381	1	1.7	3.0	2,977,895	124	4.2	3.6
2004	60,937	2	3.3	3.1	3,016,738	131	4.3	3.6
2005	61,499	6	9.8	5.5	3,056,025	166	5.4	4.4
2006	62,053	7	11.3	5.8	3,094,682	199	6.4	4.9
2007	62,572	4	6.4	4.0	3,130,943	198	6.3	5.7
2008	63,094	5	7.9	5.9	3,167,448	226	7.1	6.4
2009	63,626	7	11.0	4.4	3,204,610	230	7.2	6.5
2010	64,132	3	4.7	2.4	3,239,939	239	7.4	6.9
2011	64,583	10	15.5	9.7	3,271,415	275	8.4	7.7
2012	65,031	10	15.4	7.2	3,302,720	235	7.1	6.7
2013	65,463	8	12.2	9.5	3,332,952	232	7.0	6.3
2014	65,895	10	15.2	9.3	3,363,084	272	8.1	7.5
2015	66,335	10	15.1	6.9	3,393,814	264	7.8	7.3
2016	66,750	16	24.0	11.9	3,422,843	289	8.4	8.0
2017	67,148	8	11.9	5.9	3,450,669	294	8.5	8.1
2018	67,554	7	10.4	5.6	3,479,010	272	7.8	7.5
2019	67,952	11	16.2	10.5	3,506,853	284	8.1	8.0

### Prophet Method and Analysis on Google Collaboratory

The Prophet comprises annual and weekly seasonal effect components, a list of holidays, and a linear trend curve. The model is expressed using the following formula:


y(t)=g(t)+s(t)+h(t)+ε(t)

where y(t) denotes the observed value in the time series at time t, g(t) is the trend component at time t, s(t) is the seasonal component at time t, h(t) is the user-supplied holiday component at time t, and ε(t) is the forecast error at time t. The Prophet facilitates the analysis of different time series and filters out noise and outliers from the datasets.^22^ Then, prediction was performed using the “predict” method, and the DataFrame with the predicted values was obtained.

To evaluate the quality of the Prophet’s predictions, different metrics, such as mean square error (MSE) and root mean square error (RMSE), can be used to evaluate and compare the performance of the models:

(1) $$MSE=\frac{1}{N}\sum_{i=1}^{N}{\left(y_{i}-\hat{y}\right)}^{2}$$

(2) $$RMSE=\sqrt{MSE}=\sqrt{\frac{1}{N}\sum_{i=1}^{N}{\left(y_{i}-\hat{y}\right)}^{2}}$$

The notebook was created using the Google Collaboratory environment. The code blocks added for installing libraries were necessary for the manipulation and analysis of the data available in the spreadsheets. Among them, the “pystan” and “Prophet” libraries were used for training the data and predicting future results. Additionally, all auth (google.colab) and default (google.auth) packages were imported (“import”) to authenticate the user and obtain the necessary credentials to access their spreadsheets. In addition, we imported Gspread (a Python programming interface for manipulating Google Sheets), Prophet (Facebook), to train and predict future results), and pandas (for working with tabular data structures).

The Pandas library^23^ was used to create the DataFrames. The libraries were exported and loaded with entries (rows and columns) of the selected page with the year and corresponding mortality cases. Once the DataFrame was created, the column labeled “Year” was renamed to “ds,” while the column containing the number of cases was renamed to “y.” Next, an instance of the Prophet was created. The model was trained using the “fit” method, with the DataFrame as the input parameter. Additionally, the “make_future_dataframe” method was employed to define the future period for which predictions would be made.

### PyMannKendall Analysis on Google Collaboratory

The trend analysis of the dependent variables was performed using the non-parametric Mann-Kendall test to identify linear or non-linear temporal trends.^24^ For this analysis, the pyMannKendall package was used, a Python package that performs trend tests with non-parametric data using the Mann-Kendall algorithm and a vectorization approach to increase its performance.^20^

The pyMannKendall package was installed and imported into the Google Colab environment, along with other packages for authentication and data manipulation (e.g., Auth, Gspread, and Pandas). After authentication and selection of the appropriate worksheet, the data were loaded into a DataFrame using pandas. The relevant column (containing the number of cases for each neoplasm) was extracted and converted into a list for analysis. This list was analyzed using the PyMannKendall’s “originaltest” method, which provided results of the trend, h, P, z, tau, s, var_s, slope, and intercept parameters.

## RESULTS

### Description measures

**Figure 1:**
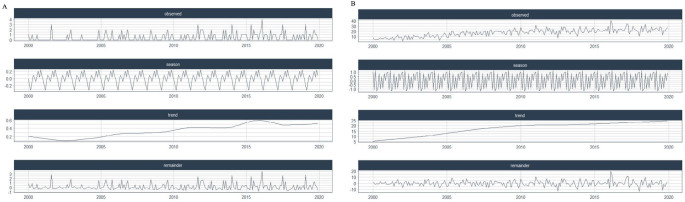
Distribution of the number of cancer-related deaths in Caicó (A) and Rio Grande do Norte State (B) using artificial intelligence.

The data obtained from DATASUS for the period 2000–2019 (before COVID-19) include the number of deaths, population, and both crude and age-adjusted mortality rates by year in Caicó and RN (**Table 1**). Additionally, the distribution of cancer mortality rates in Caicó and RN revealed the presence of seasonality and trends (**Figure 1A-B**).

### Prophet analysis for prostate cancer mortality in RN and Caicó

**Figure 2:**
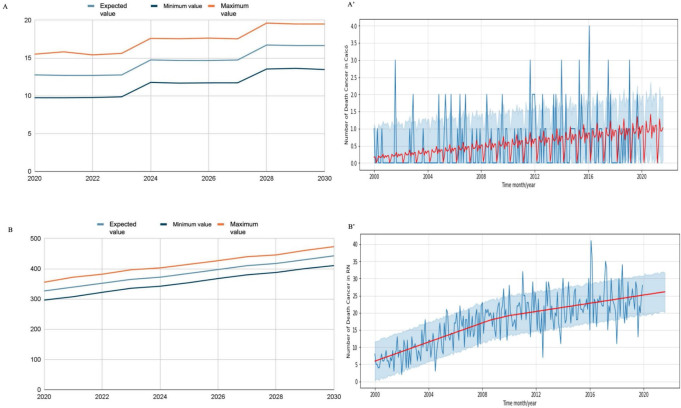
Prophet prediction analysis. A: Growth trend in mortality from malignant prostate cancer in Caicó; A’: Analysis of the number of deaths in Caicó using the Prophet model; B: Growth trend in mortality from malignant prostate cancer in Rio Grande do Norte; B’: Analysis of the number of deaths in Rio Grande do Norte using the Prophet, created using artificial intelligence

The data obtained from DATASUS until 2019 allowed the analysis of prediction between 2010 and 2019 for RN and Caicó (**Table 2**). The Prophet’s machine training predictions for RN reflect an approximation of the number of deaths when comparing the actual and predicted values. This prediction training behavior by Prophet was similar for the municipality of Caicó; however, it was more accurate in this municipality, with only a slight difference between the actual and predicted number of cases. In RN state, the analysis revealed a reduction in the predicted number of deaths in 2010–2016, an equal number of deaths in 2017, and an increase in the number of deaths in 2018 and 2019. **Figure 2A** and **B** demonstrates that the Prophet algorithm accurately captured the trend and seasonality of the data. The RMSE for the Caicó data was 2.46, indicating a good fit, while that for the RN series was 22.85, suggesting a greater variability.

**Table 2:** Machine training prediction of deaths from malignant prostate cancer performed by Prophet for the period between 2010 and 2019 for the state of Rio Grande do Norte (RN, Brazil) and the municipality of Caicó (RN, Brazil)

Year	Expected cases RN	Expected cases Caicó
2010-12-31	216	7
2011-12-31	229	7
2012-12-31	236	9
2013-12-31	249	9
2014-12-31	261	9
2015-12-31	274	9
2016-12-31	281	11
2017-12-31	294	11
2018-12-31	307	11
2019-12-31	320	11
Total	2,666	92

Thus, future predictions for 2020–2030 were established using the Prophet, noting an upward trend in the number of cases for RN (**Table 3**). The comparison of the actual data from 2010 to 2019 and the predictions from 2020 to 2030 showed an increase in the number of deaths from prostate cancer. The predicted values were approximate rather than exact.

**Table 3:** Prostate malignant neoplasm mortality growth trend – Rio Grande do Norte (2020–2030) by Prophet

Year	Expected cases	Minimum expected cases	Maximum expected cases
2020	327	296	355
2021	339	308	372
2022	352	322	382
2023	365	336	397
2024	372	342	403
2025	385	354	415
2026	397	368	427
2027	410	380	440
2028	417	388	446
2029	430	401	461
2030	443	411	473
Total	4,237	3,906	4,216

The prediction indicates an increase in the number of deaths from prostate cancer in Caicó (**Figure 2A**) and RN (**Figure 2B**). Despite this upward trend, the data revealed distinct patterns: Caicó demonstrated no growth in the number of deaths per year, while RN exhibited a consistent and steady increase in mortality rates. This behavior was illustrated with blue lines for Caicó and RN, while the Prophet’s forecast was denoted by a red curve, illustrating predictions over a 20-month period.

### Prophet analysis for testicular neoplasm mortality in RN and Caicó

No trends were observed in the number of deaths from malignant testicular neoplasia in Caicó and between 2020 and 2030.

### PyMannKendall analysis of prostate cancer mortality in RN and Caicó

The pyMannKendall (Python Library) analysis revealed a significant upward trend in prostate cancer mortality in Caicó, with a probability greater than 99% (P < 0.01). This indicates a moderate correlation; as time progresses, the number of prostate cancer deaths increases (tau = 0.586). The onset of the increase in prostate cancer deaths was determined using a variable intercept of 2.59. PyMannKendall analysis also indicated an increasing trend in mortality from prostate cancer in RN. However, the high P-value (P = 2.06) suggests that this trend is less significant, with a tau value of 0.84 and an intercept of 119.63.

### PyMannKendall analysis of testicular neoplasm mortality in RN and Caicó

For testicular neoplasia, no growth trend was detected in Caicó, with a P value of 0.6, a tau = 0.036, and an intercept of 0. Similarly, no growth trend was observed in RN, with a P = 0.43, a tau = -0.33, and an intercept of 5.5. These results indicate a reduced likelihood of an increase in the number of deaths from testicular cancer over the years (P > 0.05) and a weak correlation between the variables and tau values.

## DISCUSSION

Machine learning can aid in analyzing various health-related parameters, including monitoring of trends in disease prevalence.^10^ Therefore, we applied these Python libraries to analyze the mortality trend of two diseases that directly impact human health, with the potential for similar applications in other cities. Our analysis revealed an increasing trend in the number of deaths from prostate cancer in Caicó and RN, in addition to a stable trend in testicular cancer mortality. However, the DATASUS database does not distinguish between population groups and gender. This data gap posed a limitation in the analysis of the prediction of pathologies related to the lesbian, gay, bisexual, transgender, queer, questioning, intersex, and asexual (LGBTQIA+) population in our study.

The use of the Prophet revealed a growing trend in the number of deaths from prostate cancer in 2020–2030 in the state of RN (35.4%), with the municipality of Caicó exhibiting a similar trend (30.7%). This increase was twice as high as that observed in 2010–2019, potentially linked to the aging population, particularly among men aged over 65 years, and improvements in death certification.^25^ However, a stabilization trend was noted in Caicó between 2024 and 2027, but not in the state of RN.

Nevertheless, the mortality from testicular cancer, analyzed using Prophet and pyMannKendall, did not show an upward trend. This may be due to the low incidence of this cancer type in Brazil (3.2/100,000 inhabitants),^25^ as well as underreporting due to the limited access to healthcare services for men^26^ or incomplete data entered in Tabnet.^27^ This result differs from the projected trend of increased testicular cancer mortality in northeast Brazil (27.5%) by 2026–2030.^28^ Although this increase is smaller compared with that observed in other regions, it can be attributed to the unequal distribution of services essential for diagnosing and treating cancer patients. Our study revealed the lack of data from DATASUS regarding the special screening test for prostate cancer (CID: Z12.5), which is a key strategy for early cancer detection, both in the city of Caicó and the state of RN. Additionally, efforts targeting male health are limited, largely due to sociocultural factors related to the patriarchal system, which portrays men as bold, courageous, and confident, often neglecting their health needs.^29^

Therefore, the application of machine learning libraries can aid in situational strategic planning and decision-making at the state and municipal levels in Brazil. This approach aims to guide the healthcare team in implementing strategies that reduce the incidence of new cases of prostate and testicular cancer and minimizing the impact of these diseases on diagnosed patients.^30^ For preventive actions to be effectively integrated into healthcare, adapting health services to meet the current demands is essential. Guiding healthcare professionals through established guidelines is essential for setting goals and strategies to achieve a sustainable and resilient healthcare system that comprehensively addresses men’s health.^31^ Thus, screening tests are crucial for reducing the number of deaths from prostate cancer.

According to Cavalcanti,^32^ a comprehensive understanding of men’s health needs is crucial for the development of preventive actions.^21^ These actions are deemed successful when strategies are effectively implemented to enhance men’s access to and engagement in healthcare. Such strategies include hosting lectures, forming educational groups, providing individual consultations, and distributing brochures. According to Moura,^33^ these strategies for addressing the health/disease process ensure universal and continuous access to quality healthcare services according to the principles of universalization, equity, and comprehensiveness within the Unified Health System in Brazil (SUS).

Despite their ability to optimize data analysis, these technologies have limitations, including the need for prior knowledge of system programming, which is unknown to most healthcare professionals.^34^ Additionally, the DATASUS system has certain limitations, as it only accounts for biological sex and does not address the needs of transsexual women and transvestites in relation to prostate and testicular cancer. This gender health data gap could impact the quality and representativeness of mortality prediction.^35^ In this sense, although concrete studies on the mortality trend from prostate and testicular cancer in this population in Brazil are lacking, some authors have already highlighted the challenges that the LGBTQIA+ population faces when accessing the healthcare system.^35^ This lack of representation and visibility of transsexual bodies can lead transsexual women to feel unmotivated to seek preventive care or remain unaware of this need. They are often subjected to institutional violence, denial of rights, and neglect.^36^ This exacerbates the vulnerability of transgender women in Brazil, who have an average life expectancy of approximately 35 years.^37^ Additionally, the lack of cultural competence by healthcare providers, financial barriers, and discrimination hinder the provision of universal and comprehensive access to healthcare for this population.

Therefore, Brazil established the National Policy for the Comprehensive Health of Lesbians, Gays, Bisexuals, Transvestites, and Transsexuals (PNSI LGBT, Ministry of Health). This policy aims to improve health surveillance instruments related to gender identity and sexual orientation, thereby enhancing health information quality to monitor and evaluate health indicators for this population.^30^ However, currently, the Notifiable Diseases Information System only includes quantifiable data related to interpersonal and self-inflicted violence in its notification forms. Despite the advances made after the establishment of the LGBT PNSI, the transgender population continues to encounter difficulties in accessing healthcare services, ranging from primary care to high complexity.

## CONCLUSIONS

Machine learning models such as Prophet and pyMannKendall have accurately predicted the mortality rates of prostate and testicular cancers in the male population. However, integrating gender data, including information on transgender, into DATASUS/TabNet is essential. In addition, these results encourage the education system to create a strategy to train healthcare professionals in programming as advancements in health information technology are improving the analysis of disease trends.
